# Unraveling the cellular and molecular mechanisms of repetitive magnetic stimulation

**DOI:** 10.3389/fnmol.2013.00050

**Published:** 2013-12-17

**Authors:** Florian Müller-Dahlhaus, Andreas Vlachos

**Affiliations:** ^1^Department of Neurology and Stroke, Hertie Institute for Clinical Brain Research, Eberhard-Karls-University TübingenTübingen, Germany; ^2^Institute of Clinical Neuroanatomy, Neuroscience Center, Goethe-University FrankfurtFrankfurt am Main, Germany

**Keywords:** non-invasive brain stimulation, neurological diseases, neuropsychiatric disorders, Hebbian plasticity, metaplasticity, structural plasticity

## Abstract

Despite numerous clinical studies, which have investigated the therapeutic potential of repetitive transcranial magnetic stimulation (rTMS) in various brain diseases, our knowledge of the cellular and molecular mechanisms underlying rTMS-based therapies remains limited. Thus, a deeper understanding of rTMS-induced neural plasticity is required to optimize current treatment protocols. Studies in small animals or appropriate *in vitro* preparations (including models of brain diseases) provide highly useful experimental approaches in this context. State-of-the-art electrophysiological and live-cell imaging techniques that are well established in basic neuroscience can help answering some of the major questions in the field, such as (i) which neural structures are activated during TMS, (ii) how does rTMS induce Hebbian plasticity, and (iii) are other forms of plasticity (e.g., metaplasticity, structural plasticity) induced by rTMS? We argue that data gained from these studies will support the development of more effective and specific applications of rTMS in clinical practice.

## INTRODUCTION

Galvani (1791), an Italian physician from Bologna, was among the first to demonstrate that electrical energy drives body function. He reported that the stimulation of the frog’s sciatic nerve with a metal rod (charged with static electricity) caused contractions of the innervated muscle. Ever since this observation the question of how electrical signals in neural tissue translate into complex human behavior, both under physiological and pathological conditions, has attracted the interest of scientists and physicians around the world. Two centuries after Galvani’s famous experiments, Barker and colleagues carried out a set of analogous experiments in human subjects. To evoke muscle contractions though, Barker et al. (1985) stimulated the brain, i.e., the motor cortex, of their subjects by employing a novel non-invasive stimulation technique termed transcranial magnetic stimulation (TMS). These experiments opened several new scientific avenues and provided a means to non-invasively assess cortical excitability and function in patients with brain diseases (for review see, Hallett, 2007).

**FIGURE 1:**
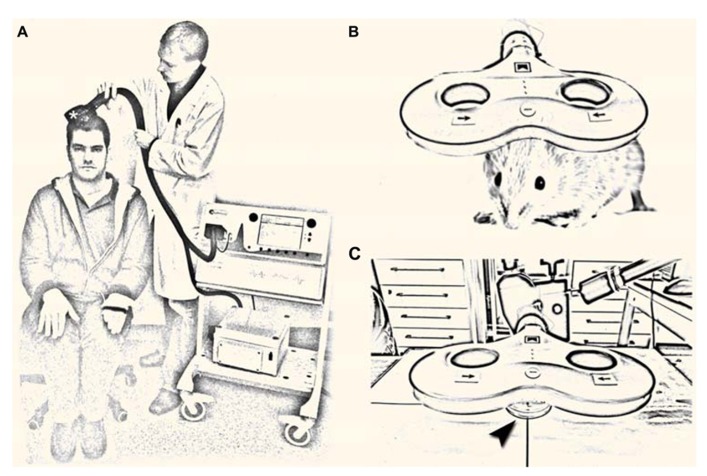
**(A)** Non-invasive brain stimulation by transcranial magnetic stimulation (TMS). A single TMS pulse of sufficient intensity applied over the primary motor cortex hand area through a TMS coil (asterisk) induces a muscle contraction in the contralateral hand and elicits a motor-evoked potential in the target muscle. Repetitive TMS (rTMS; i.e., trains of several 100 pulses) can change cortical excitability for hours beyond the stimulation period, which has driven interest toward a therapeutic use of rTMS in neurological and neuropsychiatric diseases with abnormal cortical excitability [image shows the authors; a tribute to A. Barker; (Barker et al., 1985)]. **(B,C)** Repetitive magnetic stimulation (rMS) of small animals **(B)** and suitable *in vitro* preparations [arrowhead in **(C)** points at a Petri-dish] are urgently needed to unravel the cellular and molecular mechanisms of repetitive magnetic stimulation, which remain not well understood.

Transcranial magnetic stimulation is based on the physical principle of electromagnetic induction: a strong electric current of up to several kA is discharged briefly (<1 ms duration) through a TMS coil. This generates a short-lived, but strong electromagnetic field, which penetrates the scalp and skull and induces an electric field in the underlying brain tissue. By this mechanism TMS allows for the non-invasive activation of the cerebral cortex in awake and non-anesthetized human subjects (**Figure 1A**). More recently, experimental evidence has been provided that repetitive TMS (rTMS), i.e., trains of several hundred pulses can change the excitability of the human cortex for hours beyond the stimulation period (for review see, Ziemann et al., 2008). This observation has driven interest toward a therapeutic use of rTMS in neurological and neuropsychiatric disorders associated with alterations in cortical excitability (Ziemann, 2005; Edwards et al., 2008; George et al., 2013). However, the cellular and molecular mechanisms underlying rTMS-induced neural plasticity remain not well understood. Considering data, which demonstrate a substantial inter- and intra-individual variability of rTMS-induced after-effects (Müller-Dahlhaus et al., 2008; Tecchio et al., 2008; Hamada et al., 2013), a better understanding of how TMS affects neural tissue is urgently needed.

Repetitive magnetic stimulation (rMS) of small animals and appropriate *in vitro* preparations represent suitable and highly useful experimental approaches in this context (**Figures 1B,C**). Several of these models have already been used successfully (e.g., Levkovitz et al., 1999; Meyer et al., 2009; Tokay et al., 2009; Benali et al., 2011; Gersner et al., 2011; Wang et al., 2011; Vlachos et al., 2012; Mix et al., 2013) and new important insights have been gained by these studies. Still, our knowledge of rMS-induced neural plasticity remains limited. The major focus of this perspective article is to discuss some of the open questions in the field and to illustrate how experimental approaches that are well established in basic neuroscience might help in addressing these questions. This attempt may provide a framework for future studies and could also attract the expertise of neuroscientists from other fields to join the endeavor of unraveling the cellular and molecular mechanisms of rTMS-induced neural plasticity.

## WHAT MAKES TMS DISTINCT FROM LOCAL ELECTRICAL STIMULATION?

In contrast to local electrical stimulation, i.e., a classic experimental approach to induce long-term structural and functional changes of neurons (Bliss and Lomo, 1973; Van Harreveld and Fifková, 1975; Fifková and Van Harreveld, 1977; Fifková and Anderson, 1981); for recent reviews see, e.g., Bosch and Hayashi, 2012; Colgan and Yasuda, 2013), TMS induces a widespread electric field that covers a comparatively large volume of neural tissue (up to several cm^3^; Opitz et al., 2011). This makes it difficult to predict which structures will be activated in the stimulated tissue. In recent years computational modeling has been used to estimate the electric field induced by TMS and to compute its effects on individual neurons (e.g., Rotem and Moses, 2008; Opitz et al., 2011). However, the question of which and how neural structures are activated in a given network by TMS remains unclear. Whereas local electrical stimulation can be used to activate a specific input to a neuron by depolarizing axons that are close to the stimulation electrode, it is not clear whether TMS acts strictly via the depolarization of a specific set of axons. In fact, it is conceivable that not only the afferent input, but also the target neuron itself (and other neural structures within the electric field, i.e., within the stimulated network) will be depolarized by TMS, which may generate activation patterns distinct from local electrical stimulation (c.f., Edgley et al., 1997). Thus, a central question that needs to be addressed is: which neural structures are activated by TMS during the stimulation, i.e., are specific cells or even specific subcellular compartments depolarized by TMS?

## WHICH NEURAL STRUCTURES ARE ACTIVATED DURING TMS?

**FIGURE 2:**
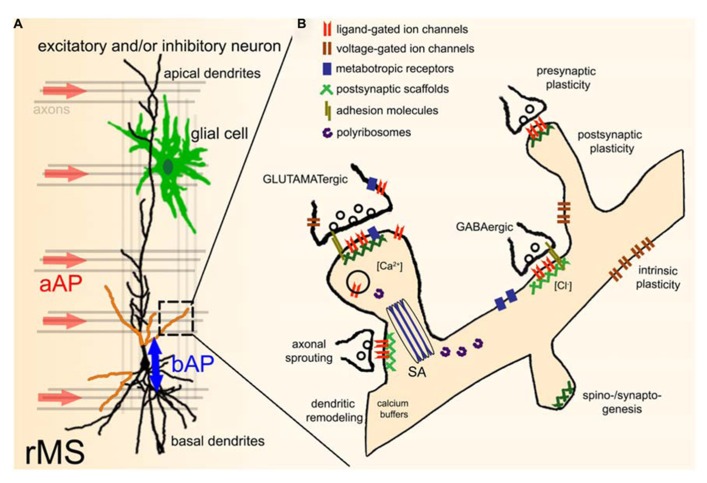
**Effects of repetitive TMS (rTMS) on single neurons within a network.** **(A)** During TMS axons with a particular orientation within the induced electric field are depolarized. This leads to anterograde propagating action potentials (aAP). In addition, TMS may also initiate backward propagating APs (bAP) in the target neuron, or even directly depolarize specific dendritic segments (indicated by orange color). TMS-effects on other neural structures, e.g., glial cells (astrocytes, microglia, oligodendrocytes) have not been investigated so far. The role of local circuits (e.g., recurrent networks, feed-forward, and feed-back inhibition) needs to be determined in this context as well. **(B)** Illustration of potential direct or indirect molecular targets of rTMS. For further details please see text [SA, spine apparatus organelle (Gray, 1959; Spacek, 1985)].

While evidence has been provided that axons are the primary target of TMS (**Figure 2A**; Basser and Roth, 1991; Basser, 1994; Rotem and Moses, 2008), it is not known whether all axons of a particular orientation within the induced electric field will be depolarized. Considering the diverse functional and structural properties of neurons, differential effects on axons of inhibitory and excitatory neurons or even specific subtypes of a class of neurons are possible. In addition, TMS may depolarize certain axons at multiple locations or produce complex spike trains by activating recurrent networks (Edgley et al., 1997), even though single TMS pulses are applied. Likewise, the depolarization of specific axons may not only lead to the induction of anterograde propagating action potentials (aAPs), but will also produce backward propagating action potentials (bAPs), which can propagate into the dendritic tree and depolarize dendrites of a target neuron (Stuart and Sakmann, 1994). Thus, specific pre- and postsynaptic structures may be activated by TMS within the stimulated network (**Figure 2A**). Furthermore, direct or indirect effects on glial cells, mitochondria, intracellular calcium stores, and calcium buffers, polyribosomes, translation/transcription factors, specific molecular complexes such as adhesion molecules, ligand- or voltage-gated channels/receptors, metabotropic receptors, postsynaptic scaffolds, and other cellular and molecular structures (**Figure 2B**) cannot be ruled out at present and thus warrant further investigation. Finally, the role of the stimulation parameters, e.g., size and geometry of the TMS coil, orientation of the coil relative to the stimulated tissue, distance from tissue, temporal dynamics of the electromagnetic field, and the stimulation protocol need to be considered (Chipchase et al., 2012). Hence, we are confronted with an enormous parameter space which can only be assessed systematically by acquiring functional data under highly controlled and standardized experimental conditions (ideally at the level of single identified cells).

Clearly, electrophysiological recordings of intact animals or suitable *in vitro* preparations represent the gold standard approach to determine the effects of TMS during stimulation. While local field potential recordings (e.g., single- or multi-electrode recordings) allow for the assessment of neural population activity (down to the level of single units), patch-clamp recordings (single- or multi-cell recordings, dual somato-dendritic recordings, recordings from astrocytes) are required to directly record TMS-induced changes in voltage, current, and firing rate of individual cells. However, these (and other suitable electrophysiological) experiments are not trivial to perform during stimulation due to the strong electromagnetic field that is generated by TMS.

An interesting solution to this problem is optical functional imaging (Scanziani and Hausser, 2009). Together with recent advances in microscopy it has become possible to visualize activity in neural tissue at high temporal and spatial resolution using proper chemical or biological sensors, even at the network level (Grewe et al., 2010). The development of novel indicators allows not only to image changes in membrane potential (Grinvald and Hildesheim, 2004; Perron et al., 2009; Marshel and Deisseroth, 2013) and monitor calcium transients (Grienberger and Konnerth, 2012; Akerboom et al., 2013; Chen et al., 2013), but to determine changes in intracellular chloride concentrations (Bregestovski et al., 2009; Berglund et al., 2011), cGMP levels (Bhargava et al., 2013), shifts in pH (Raimondo et al., 2012), and other functionally relevant molecules, both *in vitro* and *in vivo* (Smedemark-Margulies and Trapani, 2013). Furthermore, transgenic animals, (in utero) electroporation or transfection with viral vectors can be used to express genetically encoded biosensors (and/or other proteins) in selected cells, which allows for the assessment of specific cell types within neural networks (Packer et al., 2013). In addition, optogenetic approaches [e.g., the light-induced depolarization or hyperpolarization of neural structures; (Tye and Deisseroth, 2012; see also Deisseroth and Schnitzer, 2013)] can be used to discern critical elements within the stimulated tissue during TMS. In combination with pharmacological and other genetic approaches these “contact-free” techniques represent state-of-the-art approaches to identify the neural structures that are activated during TMS, and to test for their contribution to rTMS-induced plasticity.

## HOW DOES rTMS INDUCE NEURAL PLASTICITY?

During the past years experimental evidence has emerged which suggests that rTMS-induced after-effects are mediated by “long-term potentiation (LTP)-like” mechanisms (Ziemann et al., 2008). This “evidence” is based on physiological characteristics and pharmacological analogies between studies performed at the system level in human subjects and data obtained from animal models using classic LTP-experiments (see, Hoogendam et al., 2010). Yet, it has not been conclusively demonstrated at the single cell level that magnetic stimulation increases excitatory synaptic strength of cortical neurons. Using organotypic entorhino-hippocampal slice cultures we were recently able to provide experimental evidence that rMS can induce functional and structural changes of CA1 pyramidal neurons (Vlachos et al., 2012), which are consistent with a LTP of AMPA-R mediated synaptic transmission as seen after local electrical stimulation (Malenka and Bear, 2004). In contrast to classic LTP-protocols, however, in our study NMDA-R-mediated strengthening of excitatory postsynapses and an enlargement of dendritic spines were induced by a 10 Hz rMS-protocol, i.e., at comparatively low stimulation frequencies (Vlachos et al., 2012). Given the considerations above on TMS-effects during stimulation (**Figure 2A**) one may hypothesize that the induction of LTP at low stimulation frequencies is explained by the highly efficient recruitment of Hebbian-type plasticity mechanisms (Hebb, 1949) via the simultaneous (and repeated) activation of pre- and postsynaptic structures during rMS. Indeed, compartmental modeling suggests that a cooperative effect of bAP-induced dendritic depolarization and aAP-induced synaptic transmission seems sufficient to explain some of the observed rMS-induced effects on excitatory synapses of cultured CA1 pyramidal neurons (unpublished work). However, direct evidence for this “bAP-aAP theory” is currently missing. It also remains to be shown whether the precise timing of bAP-induced dendritic depolarization and aAP-mediated synaptic transmission (which will be delayed at neurochemical synapses) can lead to differential effects along the dendritic tree (for a recent review on spike timing dependent plasticity see, Feldman, 2012). Moreover, rMS-induced plasticity of inhibitory synapses and intrinsic cellular properties need to be assessed in this context (**Figure 2B**).

Apparently, these considerations do not take the specific fiber and cytoarchitecture of the stimulated network into account. It will therefore be important to compare the effects of a given r(T)MS-protocol in different brain regions (e.g., hippocampal region CA1 vs. dentate gyrus; ideally different neocortical regions). These studies can provide new important insights on the role of local circuitries in rTMS-induced plasticity. As discussed by Funke and Benali (2011), however, a major limitation of performing these experiments in the intact animal is the comparatively small brain size. The smallest coils available are still too large to selectively stimulate specific cortical regions of mice or other small animals.

## ARE OTHER FORMS OF PLASTICITY INDUCED BY rTMS

Several studies in human subjects have demonstrated rTMS-effects on cortical plasticity in the absence of detectable after-effects on cortical excitability (e.g., Hamada et al., 2008; Wankerl et al., 2010; Murakami et al., 2012). Hence, it appears important to consider rTMS-effects, which modulate the *ability* of neurons to express synaptic plasticity without (or beyond) changing the excitability of the stimulated network. The term *metaplasticity* describes this ability of neurons to change their state which influences the direction, magnitude and duration of future synaptic changes (for a recent review see, Hulme et al., 2013). It has been proposed that such “state-dependency” of synaptic plasticity could explain part of the inter- and intra-individual variability of rTMS-induced after-effects (Ridding and Ziemann, 2010). Conversely, it has been suggested that rTMS can “prime,” i.e., change the plasticity state of neuronal networks (Ziemann and Siebner, 2008). Thus, rTMS may be used as a therapeutic tool to modulate, or even restore the ability of neurons to express synaptic plasticity under pathological conditions.

A straightforward approach to test for rMS-induced metaplasticity at the cellular level is combining rMS-experiments with classic LTP- or long-term depression (LTD)-protocols (i.e., local high- or low-frequency electrical stimulation). Our work in organotypic slice cultures (Vlachos et al., 2012) has shown that the rMS-induced increase in excitatory synaptic strength of CA1 pyramidal neurons returns back to baseline 6–8 h following stimulation. It will now be interesting to test for metaplasticity by comparing the ability to induce LTP and/or LTD in cultures 6–8 h after magnetic stimulation with LTP/LTD-induction in age-and time-matched non-stimulated control cultures. A systematic evaluation of rMS-induced metaplasticity should include the assessment of (i) different intervals between rMS and classic LTP/LTD-induction, (ii) the effects of different rMS-protocols (including multiple sessions of rMS), and (iii) the molecular mechanisms of rMS-induced metaplasticity (e.g., by using pharmacological and/or genetic approaches). Likewise, rTMS-effects on synaptic tagging and capture (Redondo and Morris, 2011) and homeostatic synaptic plasticity (Turrigiano, 2012; Vitureira et al., 2012; Davis, 2013) should be evaluated. A better understanding of rMS-induced metaplasticity (and other non-Hebbian forms of plasticity) may thus help to devise rTMS-based priming strategies for optimized learning by physical exercise, language, or cognitive training in clinical neuro-restoration.

## ARE STRUCTURAL CHANGES OF NEURONS INDUCED BY rTMS?

The induction of structural plasticity, i.e., the activity-dependent re-wiring of neuronal networks is another interesting mechanism by which rTMS could assert long-lasting effects on neuronal circuits. Changes in dendritic spine/synapse numbers, dendritic remodeling, and/or axonal sprouting can all affect connectivity and thus function of neuronal networks. During the past decade advances in live-cell imaging and *in vivo* microscopy have made it possible to monitor structural plasticity for long periods of time (weeks and months) at high resolution both *in vitro* and *in vivo* (Holtmaat et al., 2009). It has become possible to monitor structures down to the level of single molecules using modern diffraction-breaking imaging techniques (for recent review see, Tonnesen and Nagerl, 2013). These new techniques open up a window to rMS-induced changes at the subcellular (e.g., mitochondria, intracellular calcium stores) and molecular level (e.g., postsynaptic scaffolds; **Figure 2B**; see also Choquet and Triller, 2013). Since live-cell imaging techniques have only been used in a single rMS-study so far (Vlachos et al., 2012), we expect a wealth of important new data on rMS-induced structural plasticity during the next years.

## HOW CAN KNOWLEDGE ON THE CELLULAR AND MOLECULAR MECHANISMS OF rTMS BE TRANSLATED INTO CLINICAL PRACTICE?

A major advantage of investigating the cellular and molecular mechanisms of rMS using appropriate animal models or *in vitro* preparations (e.g., of the hippocampus or neocortex) is the fact that various forms of plasticity (LTP/LTD, STDP of excitatory and inhibitory synapses, intrinsic cellular plasticity, metaplasticity, structural plasticity) have been studied in great detail using these models during the past decades. Data on rMS-induced neural plasticity can therefore be compared with results obtained in these *in vivo* or *in vitro* studies, which will facilitate the identification of the molecular mechanisms underlying rTMS-induced plasticity.

Since animal models of a large variety of brain diseases exist, it will be important to also study mechanisms of rTMS-induced neural plasticity in these models. Together with experiments performed under physiological conditions this knowledge will be helpful in addressing some of the current limitations/questions regarding the therapeutic use of rTMS. For example, it is not clear how rTMS-induced Hebbian plasticity, i.e., an increase in cortical excitability independent of a specific task, links to behavioral effects and could thus modulate the course of a neurological/neuropsychiatric disease. Accordingly, future studies in animal models need to address the question of whether it is rTMS-induced Hebbian plasticity and/or the induction of metaplasticity and/or interference with other plasticity-forms such as structural plasticity [or denervation-induced homeostatic plasticity (Vlachos et al., 2013)], which underlies the therapeutic effects of rTMS. These studies will also be helpful to confirm (or re-interpret) results obtained in human studies in which rTMS has been applied in conjunction with pharmacological treatments (e.g., Maarrawi et al., 2007; Fonoff et al., 2009; de Andrade et al., 2011; for review see Nitsche et al., 2012).

Eventually the knowledge gained from animal studies may be translated into clinical practice (i) by optimizing the efficacy and specificity of the stimulation to induce and/or modulate certain forms of neural plasticity with rTMS; (ii) by using knowledge about the state-dependency of rTMS-induced plasticity (e.g., by understanding the role of genetic polymorphisms, neuromodulators, metaplasticity, or homeostatic synaptic plasticity); or (iii) by combining rTMS with pharmacological interventions in order to support specific rTMS effects [an approach termed “pharmaco-TMS”; (Ziemann, 2011)]. A better knowledge of the cellular and molecular mechanisms of rMS will therefore help to optimize rTMS-based therapies and could be a step toward “personalized” rTMS-treatments of patients with neurological or neuropsychiatric diseases.

## Conflict of Interest Statement

The authors declare that the research was conducted in the absence of any commercial or financial relationships that could be construed as a potential conflict of interest.
